# Treatment of personality disorder using a whole of service stepped care approach: A cluster randomized controlled trial

**DOI:** 10.1371/journal.pone.0206472

**Published:** 2018-11-06

**Authors:** Brin F. S. Grenyer, Kate L. Lewis, Mahnaz Fanaian, Beth Kotze

**Affiliations:** 1 Illawarra Health and Medical Research Institute and School of Psychology, University of Wollongong, Wollongong, New South Wales, Australia; 2 Illawarra Health and Medical Research Institute and School of Nursing, University of Wollongong, Wollongong, New South Wales, Australia; 3 NSW Ministry of Health, Sydney, New South Wales, Australia; TNO, NETHERLANDS

## Abstract

**Background and objectives:**

People with personality disorders are prevalent in emergency and inpatient mental health services. We examined whether implementing a stepped care model of psychological therapy reduces demand on hospital units by people with personality disorder, in a cluster randomized controlled trial.

**Method:**

A total of 642 inpatients (average age 36.8, 50.5% female) with a primary ICD-10 personality disorder were recruited during 18 months baseline, then monitored during an 18 month active trial phase. In the active trial phase two equivalent sites were randomised to either treatment as usual (TAU), or a whole of service intervention that diverted people away from hospital and into stepped care psychological therapy clinics. The study design was cost neutral, with no additional staff or resources deployed between sites. A linear mixed models analysis evaluated outcomes.

**Results:**

As predicted, demand on hospital services reduced significantly in the intervention compared to TAU site. The intervention site evidenced shorter bed days, from an average of 13.46 days at baseline to 4.28 days per admission, and patients were 1.3 times less likely to re-present to the emergency department compared to TAU. Direct cost savings for implementing the approach was estimated at USD$2,720 per patient per year. Limitations included not directly comparing individual symptom changes.

**Conclusions:**

Using a whole of service stepped care model of treatment for personality disorder significantly reduced demand on hospital services.

## Introduction

Personality disorder prevalence in the community has consistently found to be 6% [1] and it is estimated that 40–50% of psychiatric inpatients have a personality disorder, with borderline personality disorder (BPD) the most prominent [2]. In relation to BPD specifically, it is estimated over 30% of persons treated in an inpatient mental health facility and over 20% of psychiatric outpatients have the diagnosis ([2–4], see [5] for a review).

Presentations of people with personality disorder to emergency or inpatient hospitals are well known to include features of significant emotional crisis, with or without a self-harm injury, or suicidal threats or attempts [6, 7]. The treatment as usual (TAU) clinical response usually involves crisis management or short-term admission for safety and de-escalation of distress, with referral to longer-term community based services [8–11]. A broad range of longer term psychological therapies are effective, particularly for BPD [12–14]. However the capacity of community mental health services and private services to offer such evidence-based treatments can be limited due to their cost and duration, typically involving at least one year of treatment, and often involving multiple sessions per week [15]. Without specialised psychological therapy programs, this patient group typically experience long waiting times for appropriate therapy, and recurrent crisis-prone behaviour and non-continuous engagement with a diverse range of therapy and other treatment options that are not optimal (ie. long-term inpatient care or off-label pharmacotherapy) [16]. Due to this and the nature of their interpersonal deficits, people with personality disorders may further escalate help-seeking behaviours, putting further strain on acute and emergency services, which may also cause ambivalence towards help provided and induce challenges for the treatment provider [17].

It is known that providing specialist long-term evidence-based approaches for the treatment of BPD have significant cost benefits compared to usual treatment [18]. However, as places in specialist long term approaches are often limited by long wait-lists, alternative pathways into suitable care need to be explored and developed that are have rapid access after crisis. Stepped care models are usually described as 'doing more with less' in that they aim to ensure the needs of the patient is balanced by the resources available [15]. Personality disorder has high prevalence, and there is significant variability in the prognosis and outcome between people with the diagnosis. Given this, Paris [19] suggests that a stepped care approach to personality disorder may be beneficial as not all patients with personality disorder need long-term care. Laporte et al [20] showed that a 12 week short term treatment can be as effective as a 24 month longer-term treatment for most but not all patients, reinforcing the benefits of stepped care approaches in the treatment of personality disorder.

This study describes and evaluates a new model designed to add a stepped model of psychological therapy in between usual crisis care and longer term treatment in the community [21]. The concept of rapid follow up interventions has been described elsewhere but mostly in the context of reducing suicide risk [22]. Initial pilot work on a similar brief intervention model was published [23] and the approach for personality disorders used here was manualised [24], and this study evaluates its impact on hospital service use.

The approach used here [21] was informed by key recommendations of Project Air Clinical Practice Guidelines and National Health and Medical Research Council Clinical Practice Guidelines (NHMRC; [25]) for BPD [13]. The operationalization of the model involves a combination of senior management and clinical leadership, training and support, and the redesign of existing service models to enhance clinical pathways to suit individual consumer need, without the need for additional resources or staff. Core to the strategy is the provision of psychological therapy, involving rapid referral to the stepped care brief intervention clinic following a crisis presentation, in order to divert from inpatient units and emergency departments (Table 1), improve the trajectory of care through the health service, and maximise use of evidence based psychological therapy approaches. Central to the provision of the stepped care model is the role of clinical staff in coordinating the flow of consumers into the brief intervention clinic, then on to engagement with appropriate longer term psychological therapy in the community.

**Table 1:** Stepped care model (intervention) with comparison to treatment as usual (TAU).

	Description	Intervention	TAU
**Step 1:** **Intake**	Identification and referral from emergency department, inpatient units, and Acute Care Teams including crisis telephone triage. **Focus:** Physical safety and crisis de-escalation.	✔	✔
**Step 2:** **Stepped Care Brief Intervention Clinic**	0–36 hours after acute presentation. Psychological safety and care planning, brief interventions with structured goal setting, connection with families and carers **Focus:** Immediate psychological therapy	✔	x
**Step 3: Psychological therapy in the community**	Evidence-based psychological therapy in the community **Focus:** Symptom remission and psychological recovery	✔	✔

With collaboration and assistance from senior management, the stepped care model was implemented in one site, referred to here as the intervention site. Two comparable geographic hospital sites within the service were given an equal opportunity to be selected as the intervention site. It was anticipated the intervention would enable a more ‘personality disorders inclusive service’ whereby systems are established to provide more effective management of patients with personality disorder in crisis. We expected that this in turn would ultimately reduce the demand by people with personality disorders on mental health inpatient hospital units and emergency departments.

## Methods

### Setting and location

This trial was carried out at one health service in New South Wales Australia. Two separate hospital sites within the same service were randomly allocated as intervention or TAU site. At the time of the study, both sites were comparable in that they were similar in size (in staffing, beds) and both had hospital mental health units servicing patients of varying acuity along with associated community outpatient services available. The two sites were separated by natural geography and landforms including a national park, meaning they operated relatively independently and the communities they served did not generally travel between sites. The primary outcomes reported here did not involve explicit written consent procedures. De-identified data was provided to the researchers for occasions of service during the initial implementation period (April 2011 to September 2012), and a baseline comparative study period (January 2009 to February 2011). The study was approved by the institutional review board in February 2011. Approval for access to electronic medical record history was also granted by the Health Services Research Governance Committees and Records Managers. The authors confirm that all ongoing and related trials for this intervention are registered. The trial was part of a state-wide health service funded initiative and the decision to evaluate this empirically was made early in the project. The protocol described in the trial registration (ACTRN12612000843853 http://www.anzctr.org.au) and study protocol accompanying this study (S1 File) is accurate and true to initial planned implementation.

### Randomisation

This study is a cluster randomised controlled trial [26, 27]. The two sites both implemented TAU for the first 18 months, meaning the intervention was delayed. This baseline phase recruited all inpatients with a diagnosis of personality disorder meeting study criteria. Then after 18 months baseline, the two sites were randomly allocated to either intervention or treatment as usual using computer sequence randomisation, and the effects were evaluated for a further 18 months by way of follow-up monitoring of the recruited participants use of health services. Management and staff from the intervention site were not blind to the condition allocated as they implemented the stepped care model.

### Standard care

The treatment as usual (TAU) was 36 months of standard care, involving usual medical care and community treatment by health care professionals, with the usual variation in services on a case-by-case basis. In this setting, it was not unusual for waitlists for long term treatment programs (e.g. Dialectical Behaviour Therapy) to be in excess of 12 months. If patients were not already engaged in treatment prior to hospital admission, they were either referred to community mental health, whereby they may have been put on a long waitlist, or referred to a community physician or private clinician, whereby participation and follow-up rates are unknown. The main difference between standard care and the intervention was that standard care had an absence of any planned stepped care approach with no brief intervention clinics available.

### Intervention

Active intervention was stepped care psychological therapy, using a whole of service guidelines based approach using an integrative relational model [21], as informed by key recommendations of the NHMRC and Project Air Clinical Practice Guidelines [13]. Psychological clinics for rapid follow-up were established at the intervention site. The model allowed the service to triage patients into brief and longer psychological therapies based on their need and readiness for intervention (Table 1). The stepped care brief intervention clinic was manualised [24], comprising one month of weekly contact. Three 50 minute sessions were with the patient, focussed on here-and-now structured psychological treatment, crisis management, safety and care planning, and provision of psychotherapy to reduce symptoms and improve psychosocial functioning. An additional session (usually between session 2 and 3) involved connection with the partner, family member(s) or other carers (usually without the patient present), to provide psychoeducation about the disorder and to facilitate positive recovery support [28]. The stepped care clinics were placed in the community mental health setting, but administratively linked to the acute crisis teams, meaning presentations to the service (e.g. emergency department, mental health inpatient units, crisis telephone line) could be identified and triaged to receive an appointment in the clinic within 0–36 hours of presentation, once any acute suicidal risk had been stabilised. The brief intervention was developed as a standard service delivered in community mental health structures by existing mental health care clinicians working in these settings. The intervention was delivered following release from hospital or the emergency department, thus any care provided in the hospital setting followed standard procedures. For community clinicians delivering the intervention, caseloads shifted from mainly long-term case management, to brief and longer treatments in a stepped care approach as described.

Further information about the brief intervention clinic, key principles, and referral criteria are available in the published manual that describes specific session outlines and supporting resources (including care plans) [24]. Following the brief intervention, patients were provided options for further community treatment by health care professionals, as available in TAU standard care. The stepped care approach allowed a responsive service to the presenting difficulties, and gave clinicians a better understanding of the patients problems and level of additional treatment required.

### Implementation of the model

Initially, implementation of the model involved a process of gaining support and assistance from senior management as well as clinical leaders within the different structures to aid in the redesign or services, to ‘champion’ the model, and to encourage relevant staff to participate in training. During the active 18 month phase, clinicians in the intervention site also had access to a series of resources including guidelines [29] and patient directed fact sheets, videos and other clinical resources to aid in treatment provided (all manuals, guidelines and resources are available at www.projectairstrategy.org). The dissemination and implementation of the model was cost neutral, using existing resources and staff.

The Project Air Strategy clinical guidelines [13, 29] describe the pathway of a consumer through the health service, from assessment and care planning, brief and longer-term therapies, and the role of inpatient and community care. They also discuss involving families and carers, as well as systemic issues for services. Staff within the services participated in specifically designed training with the aim of enhancing confidence, knowledge, and skills, and to educate on systemic changes and referral pathways. Because the intervention was implemented as a whole of service approach, participation in the training was strongly encouraged and supported by senior management and clinical leaders. Basic personality disorder training (2 hours) was delivered to 80% of all mental health clinicians working within the intervention site, and subsequent specialised training (one day) was developed and delivered to target specific aspects of working with people who have a personality disorder (e.g. stepped care psychological treatment model). Almost half (48%) of mental health clinicians within the service attended at least one full day training. The model delivered through training emphasises a compassionate, hopeful, less stigmatised response to patients with personality disorder, and also emphasises the importance of rapid implementation of structured psychological approaches following crisis. Topics in the basic level training covered engagement, assessment of urgent needs, establishment of systems of safety, orientation to services, education and support, and the development of a care plan. Subsequent full day training sessions targeted core assessment skills and more intensive application of evidence-based intervention for personality disorders treatment including the stepped model of care [21]. Specific resources used to accompany the training are also publicly available (www.projectairstrategy.org).

### Measures and inclusion criteria

All patients with mental health presentations to the hospital inpatient unit or emergency department were routinely diagnosed using a structured, trained protocol [30]. Patients were included if they (a) were aged 12 or over; (2) had at least one inpatient admission within the 18 months baseline, prior to the randomisation, and (3) had a primary diagnosis of personality disorder based on ICD-10 [31]. Administratively, these were provided based on the Australian Refined Diagnosis Related Groups model (AR-DRG; [32]) of consumer classification, whereby episodes of care are categorised and measured for the Australian Classification of Health Interventions [33]. Whole of service data for every mental health admission or emergency presentation for the 36-month study period was obtained. Data provided to the researchers was de-identified and included admission and discharge dates, demographic and diagnostic information. Same patient separations were identified using unique anonymous identifiers, grouped in chronological order and classified as either ‘baseline’ or ‘active’. The primary outcomes reported here did not involve a written consent procedure as de-identified anonymous data only was provided to researchers. The number of and length of inpatient stays and the number of emergency department presentations were the primary outcomes.

### Statistical analysis

Electronic medical record data was used to generate specific active phase and baseline indicators and used to evaluate the impact of the intervention on mental health inpatient hospital services. Specifically, we were interested in the (i) number of bed days in the inpatient unit during each phase, for each site, and (ii) number of admissions to the inpatient unit during each phase, for each site. The dataset also allowed analysis of emergency department presentations for the sample studied.

Each admission was classified as baseline or active according to the admission date, and subsequently totals were generated for the number of days spent in the inpatient unit, and number of inpatient unit admissions for the baseline and active phases. A linear mixed models approach (SPSS-21) was used to analyse the data. Specifically, a mixed models analysis was performed for each outcome variable, with covariance structure for residuals specified as ante-dependent (first order). Any pre-intervention differences on dependent variables were controlled for using this method [34]. The model was also run including gender as a covariate to determine whether any baseline gender differences had impact on the outcomes. Eleven cases were identified as extreme cases and removed from the data prior to analysis. These cases either had an extreme number of stays within the study period, or one long-term stay (300 or more days) where they were admitted within the last month of the baseline phase, and that extended through the active phase.

### Cost analysis

Cost of an inpatient bed day in a government hospital was AU$842 (see [35], Table 4.2, p. 116), equivalent to USD$881 at the time of completion of the study (AU/USD exchange $1.0464: [36]). Using this, differences in inpatient costs from baseline phase of intervention to post intervention were compared between the active intervention and control site. Cost savings to hospitals following the implementation of the stepped care approach were calculated based on the data in this study, and subsequently annualised to give the estimated yearly impact, per patient of implementing the stepped care guidelines based approach described in this paper.

## Results

**Fig 1:**
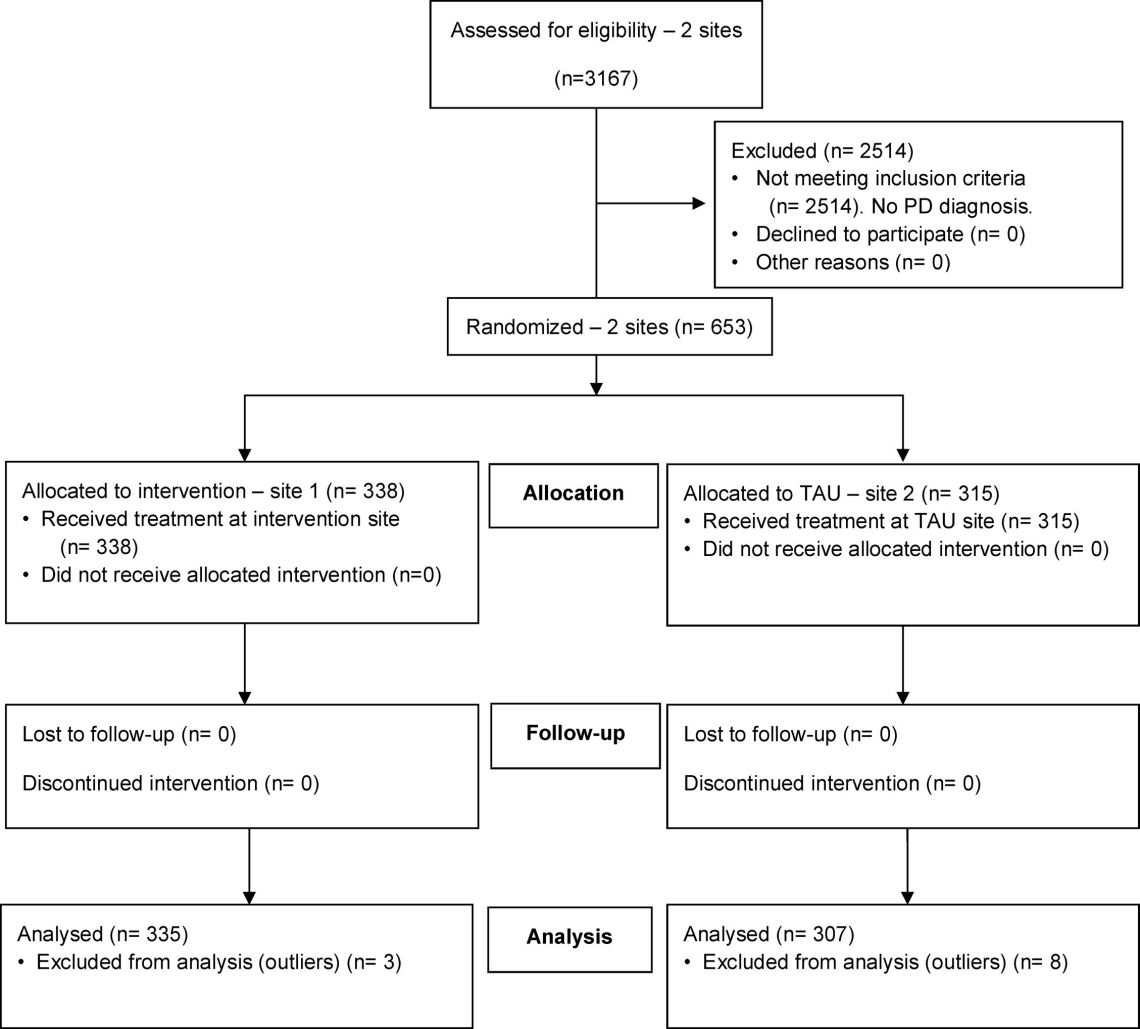
CONSORT flow diagram of patients with personality disorder in a study of hospital admissions.

Of a total 3167 patients who had been admitted to an inpatient mental health facility during the baseline phase in the intervention and TAU sites, 653 (20.6%) had a primary diagnosis of personality disorder for at least one of their stays. Participants were not included in the analysis if they were not given a primary diagnosis of personality disorder during one of their stays. Following random allocation of sites to the treatment model, 338 patients (51.8%) patients were studied who received treatment at the intervention site, and 315 (48.2%) were studied who received treatment at the TAU site. Following the removal of extreme cases (n = 11), 335 patients comprised the intervention group (52.2%) and 307 (47.8%) the TAU group (Fig 1). Patients were aged 14 through 90 years (M = 36.85, SD = 13.11). The mean age did not differ between intervention (M = 36.60, SD = 12.48) and TAU site (M = 37.12, SD = 13.78), however the proportion of females from the intervention site (46.0%) was significantly lower than the TAU site (55.4%; χ^2^ = 5.668, *p* = .017). See Table 2.

**Table 2:** Demographic characteristics of patients with personality disorder at the intervention site (n = 335) and the TAU site (n = 307).

	Intervention (n = 335)		TAU (n = 307)		*t*|χ^2^	*p*
	n | M	% | SD	n | M	% | SD		
Mean age	36.60	12.48	37.12	13.78	-.480	.632
Gender (% female)	154	46.0	170	55.4	5.668	.017
Language (% English speaking)	332	99.1	284	92.5	17.938	.000
Marital status*					.352	.553
Married/Defacto	84	25.5	70	23.5		
Single	245	74.5	228	76.5		

*note: Six patients from the intervention site have missing marital status information, and 9 from the TAU site. Thus, marital status n = 329 for intervention and n = 298 for TAU. Single includes divorced, separated and widowed).

**Fig 2:**
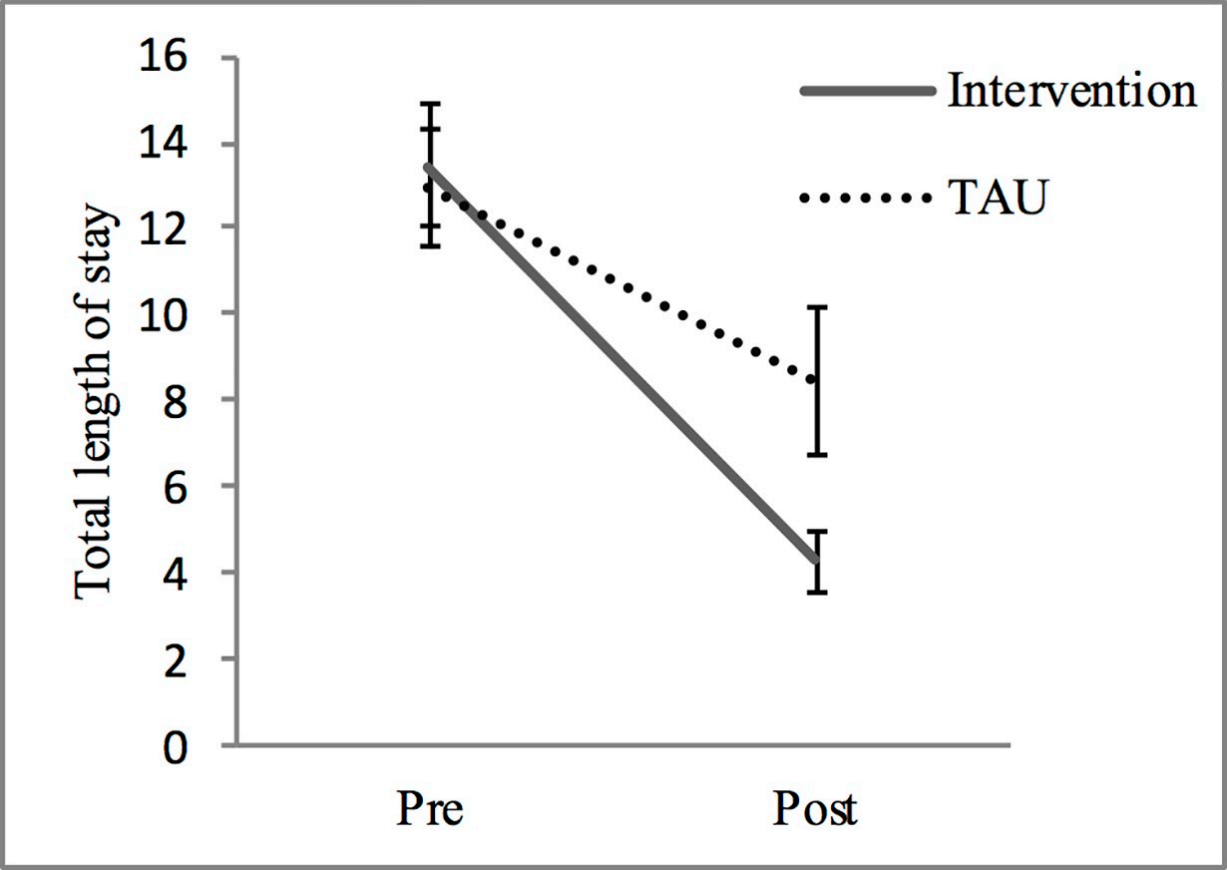
Difference in the average number of days (per patient) spent in the inpatient unit in the 18 months baseline (pre) and in the 18 months active intervention (post) of stepped care (n = 335) vs. TAU (n = 307).

For total bed days, a significant main effect for time was found (*F*(1,640) = 37.732, *p* = .000). However, when considered together, there was a significant interaction between time and study site (*F*(1,640) = 4.301, *p* = .038). Patients from the intervention site showed a significantly larger reduction in time spent in hospital in the active phase than those from the TAU, as shown in Fig 2. There were no differences between groups in total numbers of inpatient admissions across the study period (Table 3). These findings remained after controlling for gender differences between the sites.

**Table 3:** Baseline and active phase means and time*site interaction statistics for total bed days and total number of admissions to an inpatient unit (IPU). n = 642.

	Intervention					TAU						
	Baseline		Active			Baseline		Active			Time* Site interaction	*p*
	Mean	SD	Mean	SD	*d*	Mean	SD	Mean	SD	*d*		
**Days in IPU**	13.46	26.62	4.28	12.89	-.44	12.98	23.78	8.44	29.86	-.17	**4.301**	**.038**
**IPU admissions**	1.62	1.23	0.5	1.21	-.92	1.74	1.76	0.60	1.71	-.66	.006	.937

Of the patients studied, 453 had an emergency department presentation within the study period also (101 from the intervention site, and 342 from the TAU site). Using this sub-sample, we found patients from the intervention site were more 1.28 times more likely (CI = 1.17–1.40, χ^2^ = 19.980, *p* = .000) to have a reduction in ED presentations than patients from the TAU site. Specifically, 90.1% of those from the intervention site had fewer emergency department presentations, compared to only 67.5% of those from the TAU site.

### Cost analysis

The estimated mean cost per patient from the intervention site reduced from $11858 (SD $23451) during the pre-intervention phase to $3774 (SD = $11356) during the post-intervention phase. The estimated cost per patient from the control site also reduced from $11439 (SD $20950) to $7435 (SD $26309). Compared to the control site, total estimated mean cost saving following implementation of the intervention is USD$4080 per patient, equating to an estimated annual saving of USD$2720 per patient.

## Discussion

This paper describes an integrated whole of service approach to the management of personality disorders within mental health settings, and evaluates the pilot implementation in a local health district in New South Wales, Australia. A stepped care guidelines based approach [21] was designed to be integrated and adapted into existing mental health systems with collaboration and support from senior management, clinical leaders and clinicians. This study reports on the pilot roll out of the model in a health district, implemented over a period of 18 months, with comparison to a comparable site within the same health district. Data from the active intervention phase is compared with baseline data from the 18 months prior to the implementation of the intervention.

Core to the model is the provision of stepped care services and referral to suitable pathways, diverting patients from inpatient units and emergency departments, when it is not helpful. Evaluation of the stepped care approach, compared with treatment as usual showed presentations to Emergency Departments significantly reduced by 22%, and the number of days spent in the inpatient unit significantly reduced from an average of 13.46 days to 4.28 days–a significantly larger reduction than the TAU site (12.98 to 8.44 days). As a consequence, the mean cost saving for implementing personality disorder psychological treatment is estimated to be USD $2,720 per patient per year in this sample. This estimate, although only measuring the cost impact of inpatient stays, is consistent with global benchmarks outlined in a comprehensive economic evaluation review study [18], which reports an annual cost saving for psychological treatment for BPD specifically of USD $2988 per patient in total healthcare costs. Meuldijk and colleagues conclude that effectively implemented evidence based treatments for BPD demonstrate significant cost savings, and this is supported in the current evaluation of a stepped care approach.

Interestingly, we did observe a smaller reduction in hospital admissions in the TAU site also. Anecdotally, around the time of this study, and with the publication of national and international guidelines [25, 37], preference for treatment and management in the community over inpatient hospital admissions was gaining momentum. Hence, it is likely these results reflect a genuine and gradual shift in attitudes about personality disorder at the time of this study [38]. The greater improvement in the study site suggests the implementation of an intervention including emphasis on stepped care in the community and whole of service development supported and extended this shift in the field.

### Limitations and future research

This study is the first of its kind to evaluate the implementation of a stepped care model on demand for hospital services. Despite this, this study has limitations. Although the redesigning of services and staff training were limited to the intervention site only, given the size and complexity of the local health districts studied, we were unable to control conversations and distribution of resources between staff working at each site, and staff transfers between sites. Additionally, this study used anonymous data from health service records. This made it difficult to determine the exact drivers of difference between the groups, although the study had high ecological validity as service changes were made directly by clinicians.

In this study, we had access to hospital medical records. Unfortunately this access did not extend to community settings, whereby the stepped care intervention was delivered. Therefore, we were not able to evaluate the number of people referred to the stepped care intervention, nor the participation rate. The stepped care intervention however was part of a service level change, and it was expected that all patients deemed suitable for the clinic were referred to the service for an appointment. Future research is recommended to study of referral and participation rates, as well as analyses of individual patient outcomes.

Future studies might also consider the impact of a whole of service stepped care approach on different personality disorder traits or subtypes. It is possible that service impact could differ depending on severity, traits and subtype of personality disorder [39]. Similarly, it should be noted that in this study we observed substantial variation in patient age. Further study of age and its impact on frequency and duration of hospital days would allow further insight into the patterns of hospital use by people with personality disorder.

The generalizability of these findings, and applicability to other settings would depend on the practices, services available, and staff within the setting in which it is implemented. Attempts at replication of this study would have to consider the contextual factors of the site, and tailor the implementation strategy to suit. Anecdotally, the setting in this study was a typical Australian mental health service, however the generalisability of these findings to international settings is unclear. Research would benefit by implementation of the model in other settings, locally and internationally. Similarly, impact of the stepped care approach needs to be studied further at the individual level in the short and long-term.

### Conclusions

People with personality disorders comprise a significant proportion of those presenting to emergency and inpatient mental health units [3, 40]. They can be costly to health systems [18]. Psychological treatment is effective for reducing symptoms [14]. This study demonstrates that the implementation and integration of a stepped care model [21] of brief psychological clinics, using existing resources, supported and championed by senior management and clinical leaders, can result in cost savings by reduced demand on inpatient and emergency hospital services.

## Supporting information

S1 File Trial protocol. (DOCX)

S2 File CONSORT checklist. (DOCX)
