# Supplementary material for: Treatment of personality disorder using a whole of service stepped care approach: A cluster randomized controlled trial
Source: PLoS One. 2018 Nov 6;13(11):e0206472. doi: 10.1371/journal.pone.0206472 (PMC6219775; doi:10.1371/journal.pone.0206472)
Supplement: S1 File — (DOCX) [file pone.0206472.s001.docx]

##

**Randomised Control Trial Protocol**

**2011**


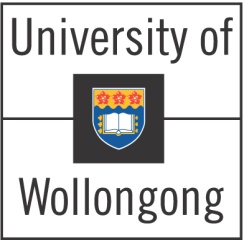

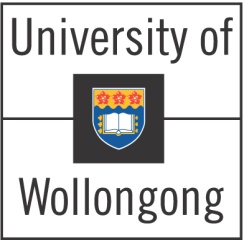

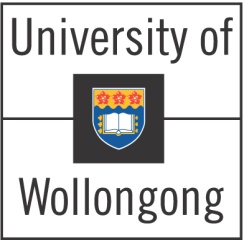

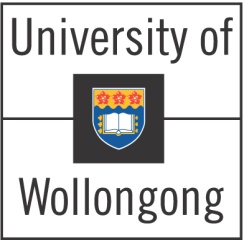

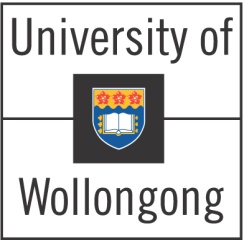


**Study Title**

**Treatment of Personality Disorders Project**

**School of Psychology**

**University of Wollongong**


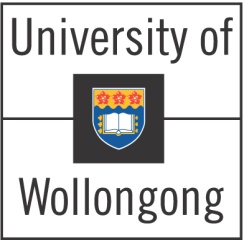

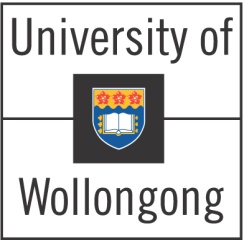


###

### Full Study Title

Treatment of Personality Disorders Project - “Treatment of personality disorders using a whole of service guidelines-based approach to improve functioning and reduce hospital use: a delayed randomised controlled trial”

### Statement of Compliance

This document is a protocol for a clinical research study. The study will be conducted in compliance with all stipulations in this protocol and the NHMRC National Statement on Ethical Conduct in Human Research (2007).

### Ethical Considerations

The clinical research study involves the evaluation of a model of care for people with personality disorders in partnership of NSW Health. The intervention is conducted by employees of NSW Health, with the University developing the model of care.

The study will be approved by the institutional review board.

The Institutional Review Board considers these applications in two steps - Human Research Ethics Committee of the University of Wollongong approves the approach to the intervention, and the Health Service provides site specific approval for access to medical records. Patients provide approval for their health records to be used for service delivery and service improvement and research, and the provision of anonymous records to researchers protects their rights to privacy with adequate safeguards overseen by the joint University-Health Service Ethics Committee (IRB).

### Amendments to protocol

Amendments to the trial protocol will be recorded in the trial registration website.

# Table of Contents

Protocol Synopsis 4

Investigators and Facilities 5

1. Study Location/s 5

2. Study Management 5

3. Funding and Resources 5

Introduction and Background 6

4. Abbreviated Background 6

5. Research Question 6

Study Objectives 7

6. Primary Objectives 7

Study Design 7

7. Type of Study 7

8. Preliminary Study Design Diagram 8

9. Number of Participants 9

10. Expected Duration of Study 9

11. Primary Outcomes 9

Study Treatments 9

12. Treatment Arms 9

Participant Enrolment and Randomisation 11

13. Recruitment 11

14. Inclusion Criteria 11

15. Randomisation Procedures 11

16. Participant Withdrawal 11

17. Handling of Withdrawals and Losses to Follow-Up 11

Procedures Schedule 12

18. Table of Procedures Schedule 12

Adverse Event Reporting 13

19. Assessment and Documentation of Adverse Events 13

Statistical Methods 13

20. Sample Size 13

21. Statistical Analysis Plan 13

Data Management 13

22. Data Collection 13

23. Data Storage 13

References 14

# Protocol Synopsis

| Title | Treatment of personality disorders using a whole of service guidelines-based approach to improve functioning and reduce hospital use: a delayed randomised controlled trial |
| --- | --- |
| Objectives | The aim of this study is to evaluate the efficacy of a whole of service approach to treatment of personality disorder |
| Design | Delayed randomised controlled trial |
| Outcomes | The primary outcome is change in hospital use measured by audit of medical record admission and discharge data. Secondary patient outcomes will be measured. |
| Study Duration | The study duration is 3 years. |
| Interventions | There is an intervention and treatment as usual arm. The intervention is a whole of service guidelines-based approach |
| Number of Participants | The study is expected to target at least 70 people |
| Population | The population involves people with personality disorder who are clients of the health service |

# Investigators and Facilities

### Study Location/s

The study involves the following locations in Australia:

*Researcher Location:*

The researchers are based at the University of Wollongong.

*NSW Health Location:*

The intervention clinicians are in the South Eastern Sydney and Illawarra Health service. The two study locations are centred on the Shellharbour hospital precinct and surrounding mental health units and services, and the St George/Prince of Wales hospital precinct and surrounding mental health units and services. The two services are similar in catchment size, staffing and clinical flow through; but are separated by a national park and natural landforms ensuring they are also serving distinct populations with separate staff servicing each precinct. This minimises contamination between sites.

### Study Management

The trial will be coordinated by an administration, research and clinical team.

2.1 Administration Team:

The administration team involves support personnel who prepare all documentation and facilitate access to research data.

2.2 The Research Team:

The research team involves the chief investigator (Professor Brin Grenyer), data manager (Kate Lewis) and a clinical research assistant (Phoebe Carter).

2.3 The Clinical Team:

The clinical team involves staff of the health department who implement actual interventions in health services lead by Service Director Assoc Professor Beth Kotze.

Brin Grenyer

Professor of Psychology

School of Psychology

University of Wollongong NSW 2522

grenyer@uow.edu.au

(02) 42213474

### Funding and Resources

This research is funded by NSW Health. The contract for the administration of the grant is through a partnership between the University of Wollongong and the Health Administration Corporation (HAC), NSW Health DOH09/33

# Introduction and Background

### Abbreviated Background

Personality disorder patients regularly present to emergency departments with self-harm or suicidal threats, and are often prematurely disengaged from treatment due to the perception that personality disorders are difficult to treat (Bateman, 2000). The clinical literature is also replete with examples of difficulties establishing rapport, high drop-out rates, and pervasive non­ compliance (McWilliams, 1994). The emotional demands on clinicians working with personality disorders are certainly well-recognised, with clinicians often reporting concern about working with this population (Brody & Farber, 1996). As a consequence, understanding and responding appropriately to personality disorder patients' needs with adequate treatment will be not only benefit personality disorder patients but may also enhance clinician competence and confidence and therefore better utilise current clinical services. Systematic investigation of current treatment programs for personality disorders will provide us with the information required to develop innovative, clinically applicable treatments for patients with personality disorders based on best research evidence. Developing our understanding of treatments will also enable clinicians to share this knowledge with patients, carers and families and will result in more collaborative and shared treatment experiences for this vulnerable population. An additional aim of the study is to distribute the knowledge acquired in the current study to mental health service systems state-wide and therefore work towards more efficacious treatments being widely available to personality disorder patients in the future. We will compare services with a specialised personality disorder service (using a stepped care model) to those without such a service across South Eastern and Illawarra Area Health Service.

### Research Question

Will a whole of service guidelines-based treatment approach be superior to treatment as usual (TAU)?

# Study Objectives

### Primary Objectives

The aim of this study is to evaluate the efficacy of a whole of service approach to personality disorders.

We will assess the impact of the implementation compared to TAU on a service level.

Clinical patients will therefore get the standard care offered in their service, however the care will vary between sites. One site will implement the intervention (whole of service approach), whereas the other will continue with standard treatment as usual.

We will study the impact of the intervention by using hospital records (inpatient admissions and emergency department presentations).

The study has two aims: (1) to assess the impact of the implementation compared to TAU on a service level, using hospital records (primary outcome), and (2) to assess the impact of the implementation on individual patient outcomes and therapist knowledge and attitudes (secondary outcomes).

# Study Design

### Type of Study

The study is a delayed randomised controlled trial. There are two treatment groups; intervention (whole of service approach) and TAU (treatment as usual) control. The study involves a baseline "treatment as usual" period for both groups, followed by one group adopting the whole of service intervention. The study is designed to determine potential superiority of the intervention over the TAU control group.

### Preliminary Study Design Diagram

**CONSORT Flow Diagram**

Randomized – 2 sites

Assessed for eligibility – 2 sites

18 month baseline

## Enrollment

Excluded (not meeting inclusion criteria)

## Allocation

Allocated to TAU – site 2

Received TAU at control site

Did not receive allocated control

Allocated to intervention – site 1

Received allocated intervention

Did not receive allocated intervention

Lost to follow-up

Lost to follow-up

Analysed
♦ Excluded from analysis

## Follow-Up

## Analysis

Analysed
♦ Excluded from analysis

### Number of Participants

It is expected that at least 70 participants will be involved in the study (35 in the intervention group and 35 in TAU control).

### Expected Duration of Study

The three year study will be in two 18 month periods - and 18 month baseline recruitment and an 18 month active intervention followup period.

### Primary Outcomes

11.1 Primary Outcome

The primary outcome involves the change in hospital use measured by audit of medical record admission and discharge data. Specifically, number of presentations to the inpatient unit, number of bed days in the inpatient unit, and number of presentations to the emergency department. Secondary analyses will involve ratings of severity of diagnostic symptoms and changes in staff confidence and skills.

# Study Treatments

### Treatment Arms

- 1. The Intervention Group

All participants attending the intervention study site for personality disorder treatment will receive the whole of service intervention. This will involve implementing a stepped care model whereby brief intervention psychological clinics are established. The role of the brief intervention clinic (Gold Card Clinic) is to transition patients with personality disorder who present in crisis for clinical services to:

(a) emergency,

(b) inpatient, or

(c) acute care services (including through the telephone triage service)

These patients will be referred, within 0-36 hours, to the brief intervention clinic attached to their acute care team for psychological therapy.

The Gold Card Clinic is a brief intervention service for people in the catchment area who have recently experienced a mental health crisis involving a diagnosed personality disorder (including self-harm and/or suicidal thoughts or behaviours).

It is one part of a whole of service stepped care approach. The goals of the stepped care model is to ensure the best and most timely use of resources based on the individual needs of the patient and their families/carers.

The GCC aims to offer an appointment within 1-3 working days of referral and offers an initial 3 sessions that focus upon identifying and addressing psychological and lifestyle factors that contributed to the crisis. An additional session for carers, partners and family members is included in the intervention.

The key aims of this intervention are to:

• provide a timely and rapid response to people seeking treatment in crisis

• provide an alternative to hospitalisation or facilitate early discharge

• provide brief interventions to help manage the client’s immediate needs

• provide brief clinical services aimed at helping the client solve their problems

• provide assessment and psycho-education to help the client understand their problems

• provide tools and strategies to help the client prevent and better manage future crises

• provide an opportunity to assess the client’s needs, including the possible need for other services where necessary

• provide an opportunity to connect with the person’s family, partner or carer where desirable

• provide treatments with an evidence-base that are effective with personality disorders

The GCC will operate during the usual opening hours of community health services (Monday-Friday, 0830-1700) and will not be available to receive referrals or meet with consumers or carers on weekends or public holidays.

Referrals

Referrals to the Clinic can be made by a range of services, including:

• Emergency Department (ED)

• Psychiatric Emergency Care Centre (PECC)

• Mental Health Intensive Care Unit (MHICU)

• Community Mental Health Team

• Community Rehabilitation Team

• Aboriginal Community Health Centre

• Early Psychosis Program (EPP)

• Acute Care Team (ACT)

Referral to crisis services

If the GCC Clinician assesses at any time that the level of risk requires an extremely urgent response they should always contact the emergency services immediately.

If the level of risk appears to require a response of any other level of urgency (i.e. low, medium or high urgency) the GCC Clinician should contact Central Intake to consider a referral to the ACT.

Discharge procedure

As a GCC Clinician is approaching the end of their work with a consumer they will bring the case to the GCC Review Meeting for discussion and discharge planning in consultation with the GCC Consultant Psychiatrist, who will ultimately authorise the person’s discharge from the service and where appropriate arrange a transfer of care to another mental health service.

As a central part of the discharge procedure the GCC Clinician will carry out a careful and collaborative consideration of further treatment and support options with the consumer and, where possible, with carers, family members and partners. This may involve a variety of actions, including but not limited to:

• Provision of resources and information about services and supports

• Signposting to specific resources, supports, services and local specialist clinicians

• Formal referrals to specific services and local specialist clinicians

• Liaison with identified local specialist clinicians to facilitate transition into longer-term treatments

• Liaison with GPs to facilitate arrangements for follow-up in primary care and access to ATAPS and Better Access Initiatives

- 1. The TAU Control

The control group involves treatment as usual (TAU) with the usual care provided by the health service for people with personality disorder seeking treatment.

# Participant Enrolment and Randomisation

### Recruitment

Participants will be clients of the health service with a diagnosis of personality disorder who have been admitted to the inpatient unit within the baseline 18 month study period.

### Inclusion Criteria

The inclusion criteria are clients having a primary diagnosis of ICD-10 personality disorder who are a mental health inpatient of the Health Service within the 18 month baseline recruitment period. They must be aged 12 or over.

### Randomisation Procedures

The study will use a computer generated randomisation sequence to allocate study sites to intervention or control. The randomisation will be performed independently from the health service and the sites.

### Participant Withdrawal

All participants meeting study inclusion will be included in this study. Whole of service hospital use data from medical records will be used as the primary data source. Participants will be withdrawn if they are considered outliers by the data control panel. Outliers include those who have been recorded as using health services in a non-standard way e.g. very rarely a patient is given different care packages such as a long term residential stay that is independent of clinical need due to pressure on housing or specific sensitivities (e.g. justice involvement).

### Handling of Withdrawals and Losses to Follow-Up

Participants who have been withdrawn from either condition will be removed from the study and documentation with regards their withdrawal will be held by the data control panel.

# Procedures Schedule

### Table of Procedures Schedule

CONTROL SITE INTERVENTION SITE

| **STUDY PERIOD** | **0-18 months**  **TAU BASELINE** | **Wash-out**  **1 month** | **19-36 months**  **TAU F/UP** | **0-18 months TAU BASELINE** | **Wash-out**  **1 month** | **19-36 months**  **Intervention**  **F/UP** |
| --- | --- | --- | --- | --- | --- | --- |
| **Recruitment of inpatients with diagnosis of personality disorder** | X |  |  | X |  |  |
| **Monitoring of Emergency use** | X |  | X | X |  | X |
| **Monitoring of inpatient bed days** | X |  | X | X |  | X |

NOTE: Washout is a one month transition between baseline monitoring (both groups) and commencement

of the stepped care model in the intervention group

# Adverse Event Reporting

### Assessment and Documentation of Adverse Events

Adverse events are likely to involve sentinel events in the health service concerning people diagnosed with personality disorder. Critical incidents and other events will be managed by (a) normal clinical governance protocols involving the involvement of senior managers and the clinical director, and (b) referral of incidents to the ethics governance unit of the health service for referral to the institutional review board. The IRB will contact researchers and seek responses to critical incidents in relation to the safe and effective conduct of the trial.

# Statistical Methods

### Sample Size

The sample size will be determined on the basis of the number of inpatients meeting the study criteria within the 18 month baseline recruitment period. All participants within the study catchment presenting for services to the health service and thus captured in the digital medical records system will be included if they meet the study inclusion criteria.

### Statistical Analysis Plan

The statistical analysis plan is to evaluate the use of health care resources across the study sites with particular attention on time x intervention site interactions. The general linear model will be employed, specifically a mixed models analysis, with the covariance structure for residuals specified as ante-dependent (first order).

# Data Management

### Data Collection

Data is routine hospital records data. The administrative team will work with hospital administrators to extract de-identified records of presentations and discharges to emergency and inpatient units where the primary diagnosis is ICD-10 personality disorder.

### Data Storage

De-identified data will be stored on a password protected university server, and therefore only those on the research team are able to access information included in the study. Original health records will be retained by the health service.

# References

Bateman, A. W., & Fonagy, P. (2000). Effectiveness of psychotherapeutic treatment of personality disorder. British Journal of Psychiatry, 177, 138-143.

Brody, E. M., & Farber, B. A. (1996). The effects of therapist experience and patient diagnosis on countertransference. Psychotherapy, 33, 372-380.

McWilliams, N. (1994). Psychoanalytic diagnosis: Understanding personality structure in the clinical process. New York: Guildford Press.
